# Characteristics of predominantly right-sided colonic diverticulitis without need for colectomy

**DOI:** 10.1186/s12893-020-00863-z

**Published:** 2020-09-14

**Authors:** Zhi Chen, Bing Zhang, Dan Wu, Ye Jin

**Affiliations:** 1 Department of General Surgery, Tongde Hospital of Zhejiang Province, Hangzhou, 310000 China; 2 Department of Anesthesiology, Zhejiang Hospital, No.1229, Gudun Road, Xihu District, Hangzhou city, 310030 Zhejiang province China

**Keywords:** Acute colonic diverticulitis, Right-sided colonic diverticulitis, Operative

## Abstract

**Background:**

In China, diverticulitis is more often located in the right colon, mainly in the cecum and ascending colon. Here we study the characteristics of acute colonic diverticulitis and compare various treatments for acute right-sided colonic diverticulitis.

**Methods:**

A retrospective analysis of 123 patients with acute colonic diverticulitis treated in our hospital from April 2013 to April 2020, including 114 cases of right-sided colonic diverticulitis, was performed. The characteristics of acute colonic diverticulitis were analyzed, and the therapeutic effects of different treatments for acute right-sided colonic diverticulitis were compared.

**Results:**

111 cases of caecal and ascending colonic diverticulitis were identified (90.2% of cases, male to female ratio 2.26:1, average age 39.6 ± 14.4 years, surgery ratio 24.3%, mean hospital stay 7.4 ± 4.3 days, recurrence rate 3.6%). Three cases of transverse colonic diverticulitis and three cases of descending colonic diverticulitis were found. Six cases of Sigmoid diverticulitis (4.9% of cases, male to female ratio 1:1, average age 67.7 ± 4.5 years, surgery ratio 33.3%, mean hospital stay 11.7 ± 5.5 days, recurrence rate 0%) were found. 13 patients underwent right-sided colonic diverticulitis resection and repair, while zero patients underwent colectomy. Abdominal drainage was performed in 15 patients with right-sided colonic diverticulitis. There was no significant difference in the length of hospital stay among the three treatments for right-sided colonic diverticulitis *(P* = 0.05). There was no significant difference in the recurrence rate among the three treatments of right-sided colonic diverticulitis (*P* = 0.358). While the recurrence rate of right-sided colonic diverticulitis was only 3.5%, relapse usually occurred within the first year following treatment.

**Conclusions:**

In our patients, right-sided colonic diverticulitis is more common in young and middle-aged patients than in elderly patients and we see a higher incidence in males. Acute right-sided complex diverticulitis is rare. While non-surgical treatment is preferred for acute right-sided uncomplicated diverticulitis, no significant difference in outcome was observed between the three different treatments we compared. Resection and repair of diverticulum or abdominal drainage can also be used to treat patients with acute uncomplicated diverticulitis.

## Background

A colonic diverticulum is a benign lesion involving a local protrusion of the colonic wall, and can arise singly or in multiples. It can occur at any position within the colon, but the characteristics of the disease vary greatly in different regions of the world. Colonic diverticulitis is considered to be rare in China. The incidence of diverticulitis is significantly higher in Europe and the United States. In these countries, diverticulitis usually occurs in the left colon, especially in the sigmoid colon. In China, diverticulitis is more often located in the right colon, mainly in the cecum and ascending colon, and the proportion in the sigmoid colon is low. In western, colonic diverticulitis is more often identified in the middle-aged and elderly, but this may not be the case in China [1, 2].

## Methods

### Patients

This article retrospectively analyzed data from 123 patients diagnosed with acute colonic diverticulitis treated from April 2013 to April 2020 in Tongde Hospital of Zhejiang Province. The diagnosis was confirmed by abdominal computed tomography (CT), colonoscopy or surgical exploration. 114 cases of right-sided colonic diverticulitis were identified. 13 patients underwent resection and repair of the right-sided colonic diverticulitis and no patients were treated with colectomy. Abdominal drainage was performed in 15 patients with right-sided colonic diverticulitis. 86 patients received completely non-surgical treatment. Cases included 85 male patients and 38 female patients (male to female ratio 2.24: 1, average age 40.0 ± 15.4 years). The study was approved by the Ethics Committee of Zhejiang Tongde Hospital.

Patients were excluded using the following exclusion criteria: 1. the patient had not yet recovered or had stopped treatment prematurely before discharge; 2. the patient’s data was incomplete or follow-up information was lost after discharge.

### Method of treatment

Patients were divided into three treatment groups. The surgical treatment group included patients who were treated surgically, by resection and repair of the diverticulum or colectomy. Following surgery this group received antibiotics and fluid replacement as treatment. In the abdominal drainage group, an abdominal drainage tube was placed by laparoscopy. The non-surgical group were treated using dietary changes, antibiotics, rehydration and other non-surgical measures.

Patients were discharged once they 1. no longer showed signs of abdominal pain or pain was obviously decreased; and 2. were fever-free for the preceding 48 h, and their inflammatory indexes such as white blood cell count (WBC) and C-reactive protein level (CRP) appeared normal.

### Data collection

The patient’s age, gender, Hinchey’s classification, diverticulum position, leukocyte index and CRP index before admission, treatment method, length of stay and postoperative follow-up of colonic diverticulum were collected.

### Statistics

SPSS17.0 statistical software was used for statistical processing. One-way analysis of variance was used to compare the mean values of multiple groups, chi-square test or fisher test was used for counting data. *P* < 0.05 was considered statistically significant.

## Results

111 patients had diverticulitis in the cecum and ascending colon (90.2%, male to female ratio 2.26:1, mean age 39.6 ± 14.4 years, surgery ratio 24.3%, leukocyte increase rate 64.9%, CRP increase rate 85.6%, 105 cases of Hinchey’s grade I and six cases of Hinchey’s grade II, average hospital stay 7.4 ± 4.3 days, the average follow-up length 33.5 ± 21.3 months, recurrence rate 3.6%); three cases in the transverse colon were found (2.4%, male to female ratio 2:1, mean age 50.0 ± 16.7 years, surgery ratio 33.3%, leukocyte increase rate 66.6%, CRP increase rate 100%, three cases of Hinchey’s grade I, average hospital stay 11.3 ± 4.0 days, average follow-up length 38.3 ± 10.2 months, recurrence rate 33.3%); three cases were located in the descending colon (2.4%, 3 males, mean age 43.3 ± 11.0 years, surgery rate 0%, leukocyte increase rate 100%, CRP increase rate 100%, three cases of Hinchey’s grade I, average hospital stay 5.7 ± 0.6 days, average follow-up length 44.3 ± 28.6 months, recurrence rate 0%); six cases in the sigmoid colon were identified (4.9%, male to female ratio 1:1, mean age 67.7 ± 4.5 years, surgery ratio 33.3%, leukocyte increase rate 66.7%, CRP increase rate 66.7%, four cases of Hinchey’s grade I and two cases of Hinchey’s grade II, average hospital stay 11.7 ± 5.5 days, average follow-up length 30.5 ± 19.2 months, recurrence rate 0%). The recurrence rate of right-sided colonic diverticulitis was 3.5%, and relapse usually occurred within one year.

Colectomy was not performed for any patients with right-sided colonic diverticulitis. Although six patients were evaluated as Hinchey’s grade II after surgery, they were assessed before surgery as Hinchey’s grade I. All six can be considered as uncomplicated diverticulitis. There are no significant differences in the three therapeutic treatments effects (Table 1). There were no complications due to intestinal leakage in both the surgical group and the group treated with abdominal drainage.

**Table 1:** Comparison of different treatments for acute right-sided colonic diverticulitis

	Abdominal drainage group(*n* = 15)	Operation group(*n* = 13)	Completely non operative group(*n* = 86)	F/χ^2^	*P*
Age(years)	34.2 ± 16.2	39.8 ± 14.6	40.4 ± 14.1	1.186	0.309
Gender				1.293	0.524
male	9	8	62		
female	6	5	24		
WBC(10^9^/L)	11.0 ± 3.8	11.4 ± 2.6	10.8 ± 3.8	0.162	0.851
CRP (mg/L)	55.9 ± 53.8	59.6 ± 52.2	55.3 ± 51.3	0.061	0.941
Hospital stay (days)	7.7 ± 3.1	11.8 ± 8.6	6.8 ± 3.0	3.655	0.050
Follow-up length (months)	35.1 ± 16.7	52.8 ± 21.1	30.6 ± 20.4	6.982	0.001
Recurrence rate	6.7%	7.6%	2.3%	1.667	0.358

## Discussion

The etiology of colonic diverticulum is currently unknown and this condition generally produces no clinical symptoms. In the United States, 4% of patients have clinical symptoms, and 15% have complicated disease [1]. In Europe and the Americas, the incidence of acute left-sided colonic diverticulitis (ALCD) is higher, while acute right-sided colonic diverticulitis (ARCD) is relatively rare, and ALCD is more common in the elderly [2]. Through a retrospective analysis of colonic diverticulitis in our hospital, we found that ARCD is more common in our patients and identified more often in males, while ALCD is rare. The onset age of ARCD in our population is younger when compared to patients in Europe and the United States. This is consistent with other reports from China [3, 4].

Because the most common type of colonic diverticulitis varies greatly in different regions of the world, there are also differences in clinical manifestations and treatment plans. Caecal and ascending colonic diverticulitis predominate in China; especially diverticulitis near the ileocecum which produces clinical symptoms similar to acute appendicitis including metastatic right lower abdominal pain, right lower abdominal fixed tenderness, and disease progression, etc. [3]. Our hospital prefers non-surgical treatment for ARED, and this conservative approach proves worthy. However, 24.6% of ARCD cases underwent invasive treatment, mainly because ARCD could not be distinguished from acute appendicitis. While all patients underwent CT examination of the abdomen prior to surgery, even experienced doctors can misdiagnose CT images. When we carefully re-analyzed the abdominal CT images after surgery, we found that it was possible to distinguish between ARCD and acute appendicitis. However, ARCD is usually characterized by acute abdominal pain, so it is difficult to rapidly diagnose before surgery. According to the WESE guidelines, ultrasonography is the imaging modality of choice for ARCD because usually patients are younger and CT imaging poses the risk of exposure to radiation [2]. Cases from our hospital support this author’s belief that CT provides advantages over ultrasound when distinguishing acute appendicitis and ARCD. The most common cause of acute abdominal pain in Chinese hospitals is acute appendicitis. Misdiagnosis of an ultrasound may result in emergency surgery. However, we assert that most cases of ARCD do not require surgical treatment, and that abdominal CT can exclude incorrect diagnoses.

Compared with acute appendicitis, we have found that the clinical symptoms of ARCD were milder, and began to resolve more rapidly after treatment. Timely and effective treatment only rarely leads to diffuse peritonitis or intestinal leakage, which inevitably cause surgeons to mistakenly think that ARCD is mild. However, in a number of surgical exploration cases, we found that the ARCD had suppurated and perforated. The affected area was partially wrapped by the greater omentum, so the clinical symptoms were mild. As mentioned earlier, the patient underwent emergency surgery because of a misdiagnosis of acute appendicitis without severe clinical symptoms. Therefore, we suspect that more patients with suppuration and perforation may be found in the non-surgical treatment group. We also found that, preoperatively, it was difficult to accurately assess whether the ARCD was perforated using CT images. A higher percentage of grade II cases were identified in the surgical group, despite a preoperative CT assessment of grade I. CT imaging appears insufficient for accurately assessing Hinchey’s classification of ARCD. The Chinese literature provides many cases of colonic diverticulum perforation, most commonly occurring in the sigmoid colon [5, 6]. This may be due to the protection offered by the greater omentum. ARCD may have been wrapped with the greater omentum before suppuration and perforation. The sigmoid colon is not easily wrapped by the omentum, and patients with sigmoid diverticulitis are generally older.

ARCD is often accompanied by increases in inflammation indicators. Some patients with mild symptoms may have a normal inflammation index. The sensitivity of CRP is relatively high, while the sensitivity of WBC is relatively low. If the inflammation index is not high, the patient can recover quickly without special treatment. MÄKELÄ et al. published a study showing that CRP > 150 mg/L is an independent risk factor for colonic diverticulitis [7]. In our study, the proportion of patients with CRP > 150 mg/L was relatively low. Simultaneously, there was no significant increase in CRP at early stages, and this value did not play an important reference role in treatment decisions. However, the CRP index is an important reference value for predicting treatment outcome. CT imaging can quickly and effectively evaluate the severity of diverticulitis, and patients with limited inflammation usually recover better.

No significant difference in patient outcome was observed between our three treatment groups. However, the conservative, non-surgical treatment was much less expensive. Therefore, we recommend a more conservative treatment approach, which is also consistent with the results of other studies [1]. At present, the most commonly used surgical method is colectomy, but that surgery produces significant trauma [8–10]. Currently, there are no detailed guidelines for the treatment of ARCD. In China, colonic diverticulitis often occurs in the cecum and ascending colon. Right hemicolectomy may be required [11, 12]. Most of the cases in our hospital underwent resection and repair of colonic diverticulum or abdominal drainage. Post surgery, there was no intestinal leakage, and the postoperative recurrence rate was low. Colectomy is not recommended for uncomplicated diverticulitis. Is it feasible to repair acute complicated diverticulitis? We lack enough patients with complicated diverticulitis to study the feasibility of diverticulectomy and repair of sigmoid diverticulitis. In our hospital, only one patient with uncomplicated sigmoid diverticulitis underwent diverticulum resection, and did not experience intestinal leakage following surgery.

In our research, we found that the rate of recurrence of ARCD was low with relapse usually occurring relapsed within the first year. Colonoscopy is not recommended during hospitalization because it may aggravate the condition. Colonoscopy is routinely recommended 2–3 months after discharge; however some patients did not undergo colonoscopy. In China, a high proportion of younger people with ARCD refused colonoscopy.

## Conclusions

In summary, ARCD is more common in young and middle-aged men. In China, ARCD is more common than ALCD, and acute right-sided complicated diverticulitis is rare. The clinical symptoms of ARCD are very similar to acute appendicitis, and can be easily misdiagnosed. While drainage of abdominal abscesses or diverticulum resection and repair are effective for acute uncomplicated diverticulitis, we recommend a more conservative, non-surgical treatment for acute uncomplicated diverticulitis. While the inflammation index was useful for predicting treatment outcome, abdominal CT proved more useful for diagnosing colonic diverticulitis.”

## Data Availability

All data generated or analysed during this study are included in this published article [and its supplementary information files].

## Abbreviations

CT: Computed tomography
CRP: C-reactive protein
WBC: White blood cell
ALCD: Acute left-sided colonic diverticulitis
ARCD: Acute right-sided colonic diverticulitis
WSES: World Society of Emergency Surgery
